# Sensitivity Analysis: A Practical Guide to Teaching

**DOI:** 10.1111/risa.70242

**Published:** 2026-04-15

**Authors:** Stefano Tarantola, Ivana Aleksovska, Mariia Kozlova, Alessio Lachi, Samuele Lo Piano, Rossana Rosati, Andrea Saltelli

**Affiliations:** ^1^ European Commission Joint Research Centre Ispra Italy; ^2^ Department of Business Studies LUT Business School Lappeenranta Finland; ^3^ Department of Medicine Saint Camillus International University of Health and Medical Sciences Rome Italy; ^4^ Faculty of Management and Economics Gdańsk University of Technology Gdańsk Poland; ^5^ Barcelona School of Management University Pompeu Fabra Barcelona Spain; ^6^ Center for the Study of the Sciences and the Humanities University of Bergen Bergen Norway

**Keywords:** global sensitivity analysis, model‐based decision making, pedagogical framework, quantitative modeling education, risk assessment, risk analysis, uncertainty quantification

## Abstract

Sensitivity analysis is the process of attributing the variability of model outputs to uncertainties in input parameters and assumptions. Why is this technique important? Which methods and concepts should be prioritized in teaching it? And what instructional strategies are best suited to different audiences? This work seeks to address these questions by drawing on the experience gained from 12 summer schools conducted by the authors since 1999, along with numerous specialized training courses delivered to academia, research organizations, and international institutions. We aim to distill these experiences into practical guidance for teaching sensitivity analysis, offering both methodological foundations and pedagogical strategies to support educators and future practitioners.

## 1 Introduction

We define “sensitive” in a way that does not appear to be widely shared in the modeling community. We call a parameter sensitive not when a change in it changes the value of an output number, but when it changes the policy conclusions we would draw from the model.'

*Groping in the Dark: The First Decade of Global Modeling*

D. Meadows et al. (1982)

This paper contributes to the methodological literature by synthesizing more than two decades of experience in teaching sensitivity analysis (SA) and translating this experience into a structured pedagogical framework for risk and uncertainty modeling. But what is SA? The term has a considerable semantic latitude. A definition offered in this journal many years ago (Saltelli 2002) reads:

Sensitivity analysis is the study of how the uncertainty in the output of a model (numerical or otherwise) can be apportioned to different sources of uncertainty in the model input.

In fact, many use SA to mean both uncertainty analysis (or uncertainty quantification)—studying the uncertainty in the output of interest—and SA proper—studying which input is responsible for it. As we shall discuss in this work, other approaches seen in the literature—for example, robustness analysis—have substantial overlap with SA.

SA can be performed using different approaches: varying a single input while holding the others constant; varying all factors simultaneously; applying small perturbations; or exploring the full range of variability. Among these, *global SA* stands out, as it systematically and jointly varies all inputs across their entire range of uncertainty through comprehensive sampling of the multidimensional input space (Saltelli 2002).

SA plays a crucial role in model‐based decision‐making, as it strengthens the robustness and reliability of decisions by informing key aspects of model development, calibration, verification, and application.

It also helps to uncover weaknesses and blind spots in modeling activities. Stakeholders at every level—from model developers to end users—can benefit from the valuable insights it provides (Saltelli et al. 2004).

In risk analysis, SA plays a critical role in identifying dominant contributors to risk metrics and in evaluating the robustness of risk‐based decisions (Borgonovo et al. 2016; Borgonovo and Plischke 2016; Clavijo et al. 2025).

Of note, a model here can be computational, logical, or mathematical, conceptual, or data‐driven. As we shall see, a model can even be a process involving several steps that lead to some form of inference.

Due to its versatility, SA has become a dynamic and expanding research field (Saltelli et al. 2021), with practical applications across nearly all quantitative disciplines (Razavi et al. 2021). Nevertheless, it remains surprisingly absent from most higher education curricula. As a result, the vast majority of practitioners today are largely self‐taught.

This work aims to lay the groundwork for teaching SA to graduate students and future practitioners by providing actionable guidelines for its instruction. We outline the essential steps for conducting sensitivity studies, offer criteria for selecting appropriate methods depending on the problem at hand, and illustrate concepts through concrete examples.

The paper is structured as follows: Section 2 discusses the importance of SA across scientific disciplines. Section 3 introduces a pedagogical framework grounded in learning theory. Section 4 presents the practical structure and content of a course on SA. Section 5 outlines expected learning outcomes, and Section 6 concludes.

## 2 Sensitivity Analysis: Why Does It Matter?

Would you go to an orthopedist who did not use X‐Ray?

*Sensitivity Analysis for Modelers*

Jean‐Marie (1996)

As noted by ecologist Charles Hall, playing with SA can be the main reason why a model is built (Hall 2020):

But the real strength of the models, in my mind at least, were in sensitivity analysis (where one could examine the response of the model to parameters or structures that were not known with precision (i.e., sensitivity analysis), and in the examination of the behavior of the model components relative to that of the real system in question (i.e., validation).

When modeling, a thorough SA can significantly improve the quality of the analysis and its communicability. The reasons for this are numerous.

SA may surprise the analyst by providing valuable, at times counter‐intuitive, results: a factor expected to be a key driver of the output uncertainty may appear as non‐influent, or vice versa (Saltelli et al. 2008); the effects of a variable on the output may go in a direction opposed to the one expected by the analyst (Norton 2015); numerical instabilities may arise that lead to code revisions; input factors may be discovered to interact, that is, they contribute to the output uncertainty via synergic effects (Saltelli et al. 1999), whereby, for example, a factor's effect can change abruptly depending on other factors (Kozlova et al. 2024). Importantly, the identification of non‐influent factors is a powerful strategy for model simplification.

SA is crucial in model calibration and validation (Pianosi et al. 2016; Campolongo et al. 2007), as it identifies parameters that have a better chance of being calibrated. Additionally, after calibration, SA can determine the overall effect of calibration uncertainty by taking as output for the analysis some measure of the quality of the calibration itself.

SA can also contribute to defending a study from being falsified, intending by this to be shown as incorrect, by enhancing the robustness (Soboĺ 1993), credibility, and transparency of the model‐based evidence (Saltelli 2002).

In scientific research, one significant challenge is the presence of degrees of freedom, whereby decisions made during data collection and analysis can significantly influence study outcomes (Breznau et al. 2022). Limited data analysis may obscure the full range of potential conclusions that could arise from different plausible methods of data processing. To address this, statisticians (Gelman and Loken 2013) have invoked the concept of the “garden of forking paths” (Borges 1998).

In recent years, the idea of a multiverse of analyses has been introduced (Steegen et al. 2016) as a means to address the issue of degrees of freedom. The multiverse analysis is a technique that systematically explores all reasonable alternatives for processing data, offering a more comprehensive and transparent view of the modeling process. Researchers employing this approach run all possible analyses using different versions of the same data and alternative methods, reflecting the variety of choices made during data collection, preprocessing, and analysis. This technique enables examination of the robustness of a particular conclusion with respect to how the data were handled. The multiverse of analyses is also referred to in the literature as the “modeling of the modeling process” (Lo Piano et al. 2022). Practical case studies (Breznau et al. 2022; Saltelli et al. 2024) have demonstrated how SA can uncover previously hidden uncertainties by efficiently exploring the multidimensional research decision spaces. In a way, one can argue that multiverse analysis is surprisingly similar to a global SA, especially when the latter takes the form of a modeling of the modeling process.

Indeed, this is not the only case of an approach to modeling uncertainty that bears a resemblance to SA, particularly global SA. Other examples are “perturbed physics ensemble” (Bellprat et al. 2012), “single‐model perturbed physics ensemble” (Stainforth 2023, 198), or perturbed parameter ensemble, or PPE (Rostron et al. 2020). The same can be said of exploratory modeling and analysis (Kwakkel and Pruyt 2013), where, in synthesis, the analysis allows variations in the inputs that are wide, regardless of their plausibility, for exploration, to answer questions of the what‐if type. Additionally, from descriptions of robustness analysis, for example, in a recent handbook on the philosophy of scientific modeling (Houkes et al. 2024), it is easy to conclude that global SA has substantial overlap with robustness analysis. This possible jumble of terminology makes the current syllabus for SA a useful clarification.

When discussing strategies to make models responsible, SA is at the top of the list of suggestions addressed to modelers. This was the case, for example, during the last pandemic of COVID‐19, where the use of mathematical models to gauge the spread of the pandemic became very visible, also to the public, where, for example, the journal *Nature* published a manifesto for “responsible modeling” (Saltelli et al. 2020). The manifesto suggested five principles for responsible modeling:

(i) minding the assumptions: being transparent about the assumptions feeding into the model;
(ii) minding the hubris: not making the model too large in relation to the data feeding into its construction;
(iii) minding the framing: being clear about the bias and worldviews of the model developers themselves;
(iv) minding the consequences: considering modeling's unintended consequences; and
(v) minding the unknowns: acknowledging that there are known and unknown elements that escape the analysis.

The manifesto also had recommendations for users; foremost, to ask for an SA before accepting the results of a model (Saltelli et al. 2020).

As discussed, these considerations do not apply only to mathematical models stricto sensu. They are also applicable across all fields of quantification, including simulation modeling, statistical inference, econometrics, machine learning, operations research, and the use of indicators of varying complexity. The course aims to illustrate these concepts via examples.

Topics that may call for a SA of model‐based inference are infinite, and infinite are the uses of modeling: SA may help evaluate the potential effects of different greenhouse gas emission scenarios on future climate conditions (Knutti and Sedláček 2013); SA can be applied to crop yield models and to optimize agricultural practices (Ruget et al. 2002); SA may enhance the reliability of complex epidemiological models (Baccini et al. 2021); in engineering, SA helps optimize designs under uncertainty (Saltelli et al. 2004); in macroeconomics, SA may help determine the impact of fiscal policy on economic forecasts, highlighting critical assumptions driving economic outcomes (Borgonovo 2007), and so on for many other cases that are impossible to list here exhaustively. A large class of its own is that of risk analysis, where SA is used to identify key risk drivers, model validation, decision‐making under uncertainty, planning of mitigation strategies, and communication.

SA plays a central role in modern risk analysis of complex safety‐critical systems (Borgonovo et al. 2016; Borgonovo and Plischke 2016; Clavijo et al. 2025). In Probabilistic Risk Analysis (PRA) models, SA is used to identify dominant contributors to risk metrics (e.g., system failure probability or core damage frequency), thereby supporting prioritization of mitigation measures and resource allocation. It also contributes to model validation by assessing the robustness of risk conclusions to epistemic and parametric uncertainty. In the design phase of safety‐critical infrastructures, such as nuclear reactors (Brown and Zhang 2016), aerospace systems (Wang et al. 2025), and energy networks (Yliruka et al. 2023), SA helps identify the parameters whose control most effectively reduces system risk and improves resilience (Marchetti et al. 2025). Historically, SA received its primary impulse from the nuclear safety community (Iman and Conover 1980; Saltelli and Marivoet 1990; Helton 1993), notably through the OECD's Probabilistic System Assessment Group in the 1980s (Sartori 2014, 95). The literature on reactor safety, for example, relative to loss‐of‐coolant accidents (and related thermal‐hydraulic risk issues, is also rich in references to the need for SA (NRC Staff 1989).

Recent developments in global SA and uncertainty quantification enable the analysis of high‐dimensional engineering simulations and complex infrastructures through variance‐based and moment‐independent importance measures, surrogate models, and computationally efficient screening approaches. These methods have been applied in nuclear fuel performance simulations (Ikonen 2016; Tsanakas and Millossovich 2016), reliability analysis, and resilience assessment of interconnected infrastructures (Liu et al. 2024), by identifying the variables that must be monitored or controlled to maintain systems within acceptable safety margins (Marchetti et al. 2025). These investigators have pioneered the use of these “grey‐box” and surrogate models to overcome the computational burden of traditional Monte Carlo simulations in safety‐critical contexts (Marchetti et al. 2025; Zio 2022).

Other investigators have extended the scope of SA beyond purely probabilistic frameworks toward a broader “risk science” perspective. Aven argues that SA must account for the “strength of knowledge” (SoK) supporting the input distributions (Aven 2020; Glette‐Iversen et al. 2023). In this view, a system is considered fragile if its risk metrics are highly sensitive to parameters where SoK is weak, regardless of the calculated probability. This shift from P‐based (probability) to (A,C,U)‐based (events, consequences, uncertainty) frameworks ensures that SA identifies not just statistical drivers, but also “knowledge‐based” vulnerabilities that could lead to unforeseen surprises or “black swan” events (Aven 2024). This multidimensional approach to SA is further exemplified by van Gelder's work, particularly in the domains of physical and maritime infrastructures. In the context of autonomous shipping, the application of SA is crucial for quantifying how variations in human and operational factors propagate through accident causation models, thereby identifying the most critical “tipping points” that compromise navigational safety. Similarly, by employing SA in the design of coastal flood defences, Van Gelder et al. (2004) demonstrate how to assess the robustness of risk‐based engineering decisions against uncertainties in meteorological and hydraulic data, ensuring that safety margins remain valid even under fluctuating environmental conditions.

Today, the convergence of Aven's foundational uncertainty principles, Zio's advanced computational architectures, and Van Gelder's practical engineering applications provides a comprehensive toolkit for managing risks in increasingly automated and interconnected technological landscapes.

A detailed timeline of SA, tracing its evolution from local to global methods, is presented in Tarantola et al. (2024).

Perhaps the most crucial reason why models need the inquisitive gaze of SA is that models are fragile. Many scholars have made this observation, starting with Pierre Duhem, whose “principle of stability” stated that a reliable model should lead to approximately the same inference when its input assumptions are approximately verified, as discussed in Fletcher (2020):

How can inferences from models to the phenomena they represent be justified when those models represent only imperfectly? Pierre Duhem considered just this problem, arguing that inferences from mathematical models of phenomena to real physical applications must also be demonstrated to be approximately correct when the assumptions of the model are only approximately true.^1^

Model volatility may result from several effects, such as the well‐known Butterfly (Schuster 1998) or Hawkmoth (Winsberg 2018) effects, referring, respectively, to the sensitivity of the model's output to boundary or initial conditions (Butterfly) or to ambiguities in model internal structures (Hawkmoth). SA practitioners are also familiar with the accumulation of parametric error in a model, known as the uncertainty cascade (Christie et al. 2011), which takes place when the model grows in size and in the number of its uncertain input parameters. At times, this fragility is compounded by the modeler's desire to build ever more accurate models (Saltelli 2019). Model fragility may surprise analysts who discover the “analytic flexibility” of a quantification when trying to replicate it in many analyst studies aimed to reproduce a statistical or econometric inference (Breznau et al. 2022), as just discussed.

Some authors note that in policy studies, model uncertainty may be artificially compressed (Funtowicz and Ravetz 1990), for the sake of achieving a usable (read: not too uncertain) result. Leamer's intimation to carefully explore the neighborhoods of the space of assumptions using global SA (Leamer 1985, 2010) is a good reminder that while models are extraordinary instruments of exploration and discovery, they are also very vulnerable to poor exploration of uncertainty.

Especially when the stakes are high and models become crucial for important decisions, this vulnerability comes with consequences. This can become evident when models are used as scapegoats to justify difficult decisions. Sociologist of science Brian Wynne notes about large modeling projects:

Whether deliberately conceived and used in this way or not, big modeling can be interpreted as a political symbol whose central significance is the diseducation and disenfranchisement of people from the sphere of policymaking and responsibility. (Wynne 1984, 311)

Revising several large computer models used in support of policy decisions, Donella Meadows, a known promoter of modeling studies in the context of the limits to growth in the seventies, has something to say about their quality management (D. H. Meadows and Robinson 1985, XIV):

The list of complaints and suggestions for improvement from practitioners in the field has not changed at all over the years, and it is still true that virtually no one is following any of the suggestions. As far as I can tell, there are no exciting new methods, no more wisdom in matching method to problem, no more imagination in depicting society, and certainly no better standards of documentation. So the examples, literature references, and conclusions we cite here are, I believe, still representative. Adding more recent examples would neither contradict nor make any of the points of the book.

These remarks are four decades old, but they reemerge periodically (Padilla et al. 2018) and become visible to the larger public at moments of crisis, such as during the COVID‐19 pandemic discussed above (Saltelli et al. 2020).

It would hence be advisable to make SA part of modeling and computer literacy training for students and future practitioners. To build a comprehensive understanding of SA, students should be encouraged to confront contradictions and uncertainties in the results of their SA. This can be achieved through group discussions, debates, and the exploration of alternative perspectives.

Teachers should also be aware that not everyone is convinced of the specific merits of SA, especially since it is not part of the syllabus taught in university courses. Despite SA having already three decades of intense research under its belt (Tarantola et al. 2024), other approaches may be more familiar to most, such as semi‐parametric models, robust estimation, or inference methods. When some model selection training was received, it would have included various types of information criteria, and not SA. Since modeling is not a discipline, and neither is SA, trained statisticians may doubt that SA can offer better results than statistics can. Practitioners of SA have experience in these positions via the reviews they receive when submitting their work to journals. The best way to assuage these concerns is via worked examples, where alternative strategies can be tested. Teachers can also demonstrate how cooperation between SA and fields such as machine learning or operations research can benefit all parties involved.

## 3 A Pedagogy for Sensitivity Analysis

SA occupies a distinctive position in risk assessment and quantitative modeling. It is not merely a computational add‐on to modeling, but a structured inquiry into how uncertainty propagates through assumptions and model structure. Teaching SA entails more than transmitting algorithms. It requires supporting learners in a shift from deterministic reasoning toward structured uncertainty interrogation. In this section, we situate SA pedagogy within established theories of adult learning and statistical education.

**Sensitivity analysis as conceptual change**. A core challenge in teaching SA is that many learners—particularly engineers, economists, and policy analysts—treat models as deterministic artifacts, equating model validation with calibration or goodness‐of‐fit. Operations research focuses on the exact representation of the optimization function and constraints, followed by optimization. SA challenges these framings by privileging exploration over optimization, and by foregrounding uncertainty, for example, when using variance‐based techniques, by decomposing output variance into structured contributions from inputs and their interactions. Looking at this through the lens of constructivist learning theory (von Glasersfeld 1989), we can consider that learners construct mental models as they receive new information. When prior mental models conflict with new evidence, conceptual change must occur. For example, SA learners must reconstruct their understanding of sensitivity, moving from one‐factor‐at‐a‐time reasoning to recognizing interaction effects and variance‐based attribution. In this respect, deterministic heuristics with counterexamples—such as functions in which first‐order effects are negligible while interaction effects dominate—serve as cognitive conflict triggers. Through guided reflection, learners reorganize their mental representation of model structure, internalizing the idea that influence is inherently multivariate and distribution‐dependent.^2^

**Threshold concepts in sensitivity analysis**. The framework of threshold concepts (Meyer and Land 2005) provides a powerful lens for understanding why SA is difficult to master. Threshold concepts are transformative, irreversible, integrative, and often troublesome. Once understood, they fundamentally alter how a discipline is perceived. Several SA ideas plausibly function as threshold concepts:

- Numerical experiments as experimentally designed,
- Variance as information rather than noise,
- Analysis of variance (ANOVA) decomposition as a structural representation of influence,
- The distinction between first order and total effects,
- Interaction as non‐additivity,
- Effective dimension in truncation versus superposition senses, and
- A modeling choice as a factor, amenable to SA.

Learners may struggle to interpret sensitivity measures, such as variance based or moment independent, not because of computational complexity, but because these indices require reconceptualizing influence probabilistically. Crossing these thresholds, practitioners begin to systematically interrogate assumptions rather than adjust parameters heuristically. Pedagogically, recognizing threshold concepts implies that instruction should expose learners to visualizations, simulations, and interpretive exercises that reinforce the take‐up of these concepts.

**Experiential learning and simulation‐based instruction**. The experiential learning cycle (Kolb 2015) includes concrete experience, reflective observation, abstract conceptualization, and active experimentation. Mapping these to an SA course yields the following steps:

1. Learners first run stochastic simulations (concrete experience).
2. They examine output variability and preliminary importance measures (reflective observation).
3. Formal ANOVA decomposition and index definitions are introduced (abstract conceptualization).
4. Learners modify model structure (active experimentation).

Simulation‐based learning resonates with modern statistical education research, which emphasizes conceptual understanding through computational experimentation rather than symbolic manipulation alone. For SA, this implies that Monte Carlo experimentation should precede formal proofs, enabling learners to observe variance patterns empirically, for example, via scatterplot or other graphical representations, before encountering their analytic decomposition.

**Adult learning and professional relevance**. Many SA learners can be PhD students, post‐docs, or mid‐career professionals. As adults, they can be problem‐centered, self‐directed, and motivated by the relevance of the problem to real‐life contexts. In risk analysis contexts, for example, this suggests that SA instruction should begin with concrete failures or controversies arising from inadequate treatment of uncertainty. Case studies in environmental risk, financial modeling, or infrastructure resilience anchor abstract methods in lived professional concerns. Participants' own models can serve as learning substrates, leveraging prior experience as an instructional resource.

**Epistemic framing: SA as model criticism**. Beyond procedural training, SA pedagogy should be framed as cultivating epistemic virtues—transparency, robustness, and critical interrogation. Teaching SA and sensitivity auditing thus becomes a model of criticism: an orientation toward identifying structural drivers of uncertainty, including those linked to the analyst's mindset and interests. This framing situates SA within the broader methodological discourse of risk analysis, emphasizing accountability and reflexivity, all the more important in policy‐relevant modeling.

Integrating these theoretical perspectives yields several design principles, such as treating SA learning as conceptual change rather than mere technical acquisition; identifying and explicitly addressing threshold concepts; structuring instruction around experiential simulation cycles; and anchoring instruction in authentic industrial, managerial, societal, environmental, or political problems.

## 4 Content of a Course on SA: What to Teach

I have proposed a form of organized SA that I call “global sensitivity analysis” in which a neighborhood of alternative assumptions is selected and the corresponding interval of inferences is identified. Conclusions are judged to be sturdy only if the neighborhood of assumptions is wide enough to be credible and the corresponding interval of inferences is narrow enough to be useful.

*Sensitivity Analyses Would Help*

Leamer (1985)

We provide in Table 1 three possible course formats: short, medium, and long, corresponding to one, two, and four ETCS credits.^3^ Under other methods, we imagine teaching new developments in SA, such as methods based on optimal transport (Borgonovo et al. 2025), the research bridging SA with feature importance used in machine learning (Antoniadis et al. 2021), or linking local and global (glocal) methods (Borgonovo et al. 2016). A summary of the key steps should include the elements in Table 1 for all formats of the training.

**TABLE 1:** Topics for the course.

Topic	Key source	Format
Introduction to UA and SA	Saltelli et al. (2008)	All
Experimental design for SA	Becker and Saltelli (2015)	All
Variance‐based methods I	Saltelli et al. (2008)	All
Variance‐based methods II	Saltelli et al. (2008)	Medium
Moment‐independent methods	Borgonovo (2007)	Long
Effective dimensions	Kucherenko et al. (2011); Owen (2013)	Long
Non‐independent input	Kucherenko et al. (2011)	Long
Meta‐modeling	Sudret (2008)	Long
SA via machine learning	Antoniadis et al. (2021)	Long
Shapley values	Owen (2014)	Long
Sensitivity auditing	Saltelli (2025)	All
Sociology of models	Morgan (2012)	Long
SA for dummies	Kozlova (2025), Helton (1993)	All

**Definition of the scope of the analysis**. Students should be introduced to literature, emphasizing that effective SA must begin with a clear definition of the scope and context. This foundational step ensures that the purpose of the analysis and the outcomes of interest are precisely articulated. The study's context can vary depending on objectives, including

- Prioritization: Focusing on key inputs for deeper exploration,
- Model simplification: Fixing certain inputs to reduce the size of a model,
- Identifying critical input regions: Locating influential areas in the input space,
- Data evaluation: Examining the characteristics and limitations of available data, and
- Decision support: System behavior exploration.

These objectives ultimately serve two complementary purposes: model development and system exploration. The former helps refine the model so it is better aligned with the realities of the case, while the latter reveals the implications the model carries for decisions or actions it is meant to support.

Importantly, the specific outputs of interest must be explicitly defined and differentiated from other potential model outputs. This point is crucial and was well expressed already in the 1980s by the first “modelers of the world” (D. Meadows et al. 1982) in the quote at the beginning of our introduction.

This means that if the model is being used for a new purpose its sensitivities should be reassessed (Edmonds et al. 2019), that is, a single SA cannot serve all purposes for a given model. In reality, when a model is applied to new contexts, a fresh SA is required. In turn, the new SA may show that the model is inadequate to answer the question posed to it, and hence call for developing a revised model version, or for reframing the questions being addressed through the analysis.

Different analytical settings require distinct considerations, and students should recognize the boundaries and limitations inherent in each approach. To make an example, SA can be specifically tailored to identify the most important factor worth further data collection, or simplify the model by reducing non‐influential factors, or identifying a subset of factors responsible for a given share of the total variance of the output, and so on. This understanding will help them connect specific SA objectives with appropriate methodological choices.

**Identification of inputs and their classification**. Students must be guided to systematically identify and characterize uncertainty in model inputs. A key learning objective is to encourage a comprehensive yet judicious approach: they should cast a wide net to include as many uncertainty sources as relevant, but this must be tempered with careful judgment in defining the extremes and statistical distribution shapes (e.g., ranges, correlations, or probability density functions). Note in this respect that while uncertainty is most often underexplored, it can, in specific cases, be overestimated instrumentally, as when in a policy controversy one party wants to show that a given course of action (e.g., limiting the exposure to a pollutant) yields too uncertain estimates to justify the costs (Saltelli et al. 2013; Michaels 2008).

A critical aspect involves the use of triggers—discrete inputs that allow the model to dynamically select alternative formulations, scenarios, or policies at runtime; staying with the metaphor of the garden of forking paths discussed above, a trigger can be used to decide whether to take one path versus another in the garden: a data imputation strategy versus another, whether include or not a control, the form of a regression model, the use of a specific formula, among the different that are reported in the literature, for example, for evapotranspiration, and so on (Puy, Sheikholeslami, et al. 2022).

Students should be prepared for the possible use of models in a participatory context. Here, the modeling activity should become an instrument that the various parties use to chart the impact of different perspectives and beliefs. This kind of learning can be facilitated by the study of existing cases, such as Lane et al. (2011) and Nabavi (2025). Working with time series and spatial maps as both inputs (e.g., climate projections, land‐use data) and outputs (e.g., risk maps, impact forecasts) is also useful. This includes understanding how temporal and spatial dependencies influence uncertainty propagation.

It is important to stress the difference between statistically independent inputs (e.g., parameters that vary without influencing each other) and dependent inputs (e.g., correlated variables such as temperature and humidity). Emphasizing this distinction helps students avoid methodological errors in SA and ensures robust interpretation of results.

**FIGURE 1:**
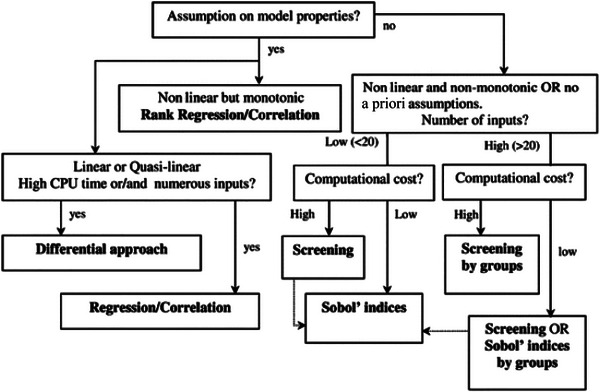
Decision diagram for the choice of a SA method (from de Rocquigny et al. 2008).

**Choice of method to be applied**. Choosing an SA method involves balancing several aspects. For example, analysts might want high accuracy in their sensitivity measures, but this often requires significant computational resources. As a result, SA techniques balance the computational effort required for a single model run with the total number of model runs needed to achieve reliable results (Iooss and Saltelli 2016; Iooss and Lemaître 2015). Selecting an appropriate SA method will also critically depend on the specific objectives of the analysis. Different methods are better suited for different situations. Here are some elements to consider when selecting a method:

- What does one want to find out? For example, identify the most important factors, or understand how they interact with each other, or identify those who are the least important, to simplify the model or the analysis?
- How much analyst time and computer resources are available? Should one use for a screening method or a more thorough quantitative one (Campolongo et al. 2011)?
- Related to the above, how many input factors are being considered? Screening methods are better suited to deal with a large number of factors.
- Can certain features of the model, such as linearity or monotonicity between inputs and outputs, be anticipated?
- Is the sample already available (data‐driven analysis) or should it be designed ad hoc (design‐driven analysis)?
- Who is the recipient of the analysis? Some methods are simpler than others and can be explained in plain English. This is important when the recipients of the analysis are not themselves trained in STEM (science, technology, engineering, mathematics) disciplines.

(See also the decision tree in Figure 1 and Table 2 for guidance on the method selection.)

**TABLE 2:** This table provides an overview of the key features of the primary SA methods, focusing on representative approaches. It does not include more specialized techniques such as moment‐independent SA or variogram analysis.

	Linear regression	Rank regression	Screening	Variance based	Meta‐modeling
Number of factors	<100	<100	<100	<25	<50
Computational cost	100/1000	100/1000	∼10k	∼50k	100/1000
Cope with non‐linear models	No	Yes	Yes	Yes	Yes
Cope with factors' interactions	No	No	Yes	Yes	Yes

*Note*: The values presented in this table are derived from established practitioner practices. Note that while not a SA method per se, meta‐modeling is a powerful tool for constructing surrogate models. These surrogates, which execute rapidly (often in negligible time), enable efficient variance‐based SA even for problems involving up to 50 input factors. For problems with approximately 100 input factors, screening methods are recommended as a preliminary step. These identify and eliminate non‐influential factors, narrowing the focus for subsequent variance‐based SA. A Monte Carlo sample of input factors can simultaneously support: (i) linear and rank regression to assess linear or monotonic relationships between inputs and outputs, and (ii) meta‐modeling to construct a surrogate for deeper structural analysis. This combined strategy facilitates an initial understanding of model behavior and informs the decision to apply variance‐based methods for more rigorous, quantitative sensitivity measures if required.

**Generation of the input sample**. Here, the students learn about the importance of a well‐designed sample and how the convergence of the analysis depends upon it. Random, Latin Hypercube, and Quasi‐Random sampling schemes are taught here.

The input sample must allow a full investigation of the input space. Its size should be defined consistently with the available computational time, the model features, and the required level of accuracy of the sensitivity estimates. Tools to monitor the convergence of the output are also given here. The sample of the input and outputs (I/O sample) over all simulations constitutes the input for the SA.

**Application of the method and reporting of the results**. Applying the selected method to the I/O sample, the sensitivity measures are obtained. The SA process may be complex, and SA results should be reported comprehensively for the final users to understand what has been learned and the limitations of the exercise. It is important to go beyond reporting the sensitivity indices and to visualize input–output relationships identified as influential. A single index may be misleading when an input's effect varies across the output space. Techniques for multidimensional data visualization (Kozlova et al. 2024; Piccolotto et al. 2023) or recomputing indices for subsets of data (Borgonovo et al. 2024) can reveal these variations and ensure that the recommendations from SA remain well aligned with the underlying patterns.

As mentioned above, it matters if the recipient of the analysis is a policymaker, a regulator, a quality manager, a researcher, or other lay stakeholders. The task could be particularly challenging for the teacher because students' own technical training may lead them to focus on that analysis and not on who needs to understand it.

Some of the points just illustrated can be summarized in a checklist:

- Explore the input space globally, not one factor at a time,
- Do not sample just parameters and boundary conditions, but also structures, frames, and problem definitions,
- Justify the choice of the method—what is meant by importance in the given context?
- Do not ever run the model just once. Use a Monte Carlo driver for uncertainty and SA also at the stage of model building (there is always one more bug to be discovered),
- Do not vary each uncertain factor by a fixed percentage. This would imply that either all factors are equally uncertain, which is rare, or that the analysts did not assess the plausible uncertainty range of each factor, leading to a perfunctory SA,
- Whatever SA method is chosen, results need to be explained in plain English, and
- Visualize the most critical effects using multidimensional data rendering to expose the shape of interactions.

**Summary of sensitivity analysis resources and tools**. A range of software tools and learning resources is available today that facilitates the application of different SA methods in a variety of languages. Table 3 offers a selection of these resources, including their academic references, online repositories, and open‐source libraries. They are categorized by functionality and accessibility.

**TABLE 3:** Software tools for sensitivity analysis.

Category	Resource/tool	Key features	Access/URL	Ideal users/application
General resources	R Handbook	Foundational resource for SA principles and methodologies	Da Veiga et al. (2021)	Researchers seeking foundational knowledge
	SAMO Conference Material	Advanced discussions, publications, and methodologies	SAMO Website	Researchers and educators in SA
	European Commission (JRC)	Frameworks, SimLab software, and global SA tools	JRC Website	Practitioners of European Commission tools
	Réseau Mexico	Resources and MTK (Mexico Tool Kit) for collaborative SA	Réseau Mexico	Collaborative SA projects using MTK
Software tools for SA	SimLab	Freely accessible for global SA with online tools	SimLab	Educational use and SA projects
	OpenTURNS	C++/Python library for uncertainty quantification	OpenTURNS	Statistical modeling and risk assessment
	UQLab	MATLAB and Python‐based framework with detailed documentation	UQLab	MATLAB and Python researchers and educators
Python libraries	SALib	Python library for SA methods like Sobol', FAST, and PAWN	SALib	Python programmers and educators
R packages	sensitivity	Package for variance‐ and screening‐based SA methods	sensitivity	Students and professionals in R
	sensobol	Tools for variance‐based sensitivity indices in R	sensobol	Researchers focusing on variance‐based SA
	MTK (Mexico Tool Kit)	Workflow‐oriented toolkit for executing SA methods	MTK	Collaborative R projects
Specialized tools	GEM‐SA	Gaussian process‐based SA for intensive models	GEM‐SA	Advanced SA applications
	MUCM Project	Educational materials for SA and uncertainty analysis	MUCM	Educators and researchers
Spreadsheet tools	SA Excel Add‐In	Free Excel Add‐In for sample‐based SA	Excel Add‐In	Beginners using Excel

These tools offer a variety of features tailored to different levels of complexity and user expertise, from introductory options like the Excel add‐in for fundamental SA to advanced libraries such as OpenTURNS and GEM‐SA for more complex model evaluations. Figure 1 is a simplified and schematic diagram to orient the analysis in the decision about which method to use.

**From global SA to sociology of quantification**. A critical sociological perspective should also be incorporated: any quantitative analysis involves both technical quality and normative dimensions. The latter requires awareness that models may inadvertently omit stakeholder perspectives or visions of the problem (Jasanoff 2003).

The dependence of numbers upon the context—historical, social, and political—in which they are produced is well known to sociologists and historians (Porter 1995; Desrosières and Desrosières 2011; Hacking 1990), be it that the numbers of different disciplines, such as, for example, physics or economics, obey different laws and are subject to different challenges (Mennicken and Espeland 2019). When the output from a model feeds into a context where policy disagreement might be expected, SA can be extended to a broader pool of uncertainties. This is the realm of sensitivity auditing (SAUD; Saltelli et al. 2013), which extends model quality analysis by questioning the objectives, purposes, and biases of both model developers and problem owners.

SAUD targets a more general class of issues than GSA (see Edward Quade's “pitfalls of analysis and modeling”; Quade 1980). SAUD has analogies with NUSAP, a pedigree for the quality of quantitative information developed in Funtowicz and Ravetz (1990). The acronym stands for number, units, spread, assessment, and pedigree, where spread can be, for example, an uncertainty bound, assessment refers to the quality of the study, and pedigree refers to the quality of the team performing it. These last attributes are intended as resulting from the work of an extended peer community (van der Sluijs et al. 2005). “Sensitivity auditing, [...] is a wider consideration of the effect of all types of uncertainty, including structural assumptions embedded in the model, and subjective decisions taken in the framing of the problem,” according to the EU guidelines for impact assessment (Commission 2023, 563).

It extends SA to normative and policy issues when a quantification feeds into policy, with a seven‐point checklist:

- check against the rhetorical use of mathematical modeling;
- adopt an “assumption hunting” attitude;
- detect garbage in garbage out, in the extended definition of (Funtowicz and Ravetz 1990);
- find sensitive assumptions before these find you;
- aim for transparency;
- do the right sums;
- focus the analysis on the key question answered by the model, exploring holistically the entire space of the assumptions (verbatim from Saltelli et al. 2013).

Sensitivity auditing is also described in the guidelines of SAPEA (Science Advice for Policy by European Academies) (SAPEA 2019, 88–89). Applications of sensitivity auditing that can be used as an illustration in the teaching are Lo Piano and Robinson (2019), Sheikholeslami et al. (2022), Lo Piano et al. (2024), and Lo Piano and Saltelli (2025).

**The GitHub Repository**.

Models can corroborate a hypothesis … Models can elucidate discrepancies with other models. Models can be used for sensitivity analysis ‐ for exploring ‘what if’ questions ‐ thereby illuminating which aspects of the system are most in need of further study, and where more empirical data are most needed.

*Verification, Validation, and Confirmation of Numerical Models in the Earth Sciences*

Oreskes et al. (1994)

A set of representative test cases is provided in the GitHub repository https://github.com/Sensitivity‐Analysis‐for‐Model‐Output/HowToTeachSensitivityAnalysis

These cases can help put the concepts covered into practice. Table 4 summarizes the test cases and explains the rationale behind their selection.

**TABLE 4:** Table with main test cases.

Case	Key source	Rationale
Probabilistic weather forecasting	Kerin and Engler (2020), Palmer (2019)	An end‐to‐end Lorenz‐96 workflow demonstrating how perturbations in meteorological initial conditions propagate through the model and affect forecast reliability, quantified via Sobol' sensitivity indices of the Spread‐Skill Ratio score
Investment profitability	Kozlova et al. (2024)	A simple illustration of an ubiquitous interaction effect necessitating global SA
Carbon footprint	Kozlova et al. (2024)	A demonstration of how one factor switches the direction of influence depending on another factor
Steel structures reliability	Ahola et al. (2024)	An example of varying importance of several factors, stressing the need for supporting SA with visualization
Portfolio modeling	Saltelli et al. (2004)	A simple illustration of how global SA can find use in finance
Infection dynamics	Puy, Beneventano, et al. (2022)	To show how increasing the complexity of the infection dynamic equations leads to cases of increasing complexity and effective dimension

**Student's assessment**. The teacher needs to select assessment strategies for evaluating student understanding and progress in SA, such as project‐based assignments, quizzes, presentations from groups of students, and interactive discussions. These assessments should focus on both the theoretical concepts and practical applications of SA and feed into a dynamic adaptation of the course to make good use of lessons learned.

## 5 Learning Outcomes

**Short course version: one ETCS credit**. Upon completion of the short version of this course, students will be able to

1. *Understand key concepts*: Distinguish between uncertainty analysis and SA, recognizing their distinct purposes and applications in modeling and decision‐making processes.
2. *Design numerical experiments*: Appreciate the importance of careful experimental design to systematically explore multi‐dimensional factor spaces and conduct effective numerical SA experiments.
3. *Select best practices*: Choose appropriate variance‐based SA methods, tailoring their selection to suit diverse problem settings.
4. *Recognize method limitations*: Understand the limitations of local SA techniques and the significance of employing global methods to capture a broader range of model behaviors.
5. *Define importance of input factors*: Comprehend the necessity of clearly defining the “importance” of input factors within the context of SA to yield meaningful results.
6. *Explore test cases*: Gain experience with a variety of test cases that offer inspiration for applying SA to their own research.
7. *Leverage software tools*: Acquire knowledge of software available in various programming languages for conducting SA.
8. *Embrace Monte Carlo methods*: Internalize the concept of developing and refining models within a Monte Carlo framework to manage uncertainty.
9. *Enhance robustness of the analysis*: Recognize the value of SA in increasing the robustness of analyses, including the potential for modeling the modeling process itself and conducting sensitivity auditing.

**Medium course version: Two ETCS credits**. The teacher can tailor this format of the course to her/his objectives and interests, selecting from the medium and the long course specifications.

**Long course version: Four ETCS credits**. In addition to the outcomes listed in the short course version, students completing the long version of the course will

1. *Expand methodological knowledge*: Learn to employ an expanded range of SA techniques, including moment‐independent methods, strategies for sampling from reduced parameter spaces, and approaches for handling non‐independent variables.
2. *Develop coding proficiency*: Gain hands‐on experience coding SA methods directly in one or more programming languages, enhancing their computational skills and understanding of algorithmic implementation.
3. *Broaden disciplinary applications*: Be exposed to a wide array of test cases from various disciplines, enabling them to appreciate the versatility of SA across different fields and problem contexts.
4. *Deepen Analytical skills*: Develop the capability to analyze and interpret SA results critically, and to communicate these findings effectively to stakeholders with varying levels of technical expertise.

## 6 Conclusions

Teaching SA has broad implications for the scientific community, education, and the fields of quantification and modeling. The authors of this work have repeatedly encountered cases of quantification in which an honest SA would have been essential. The epistemic authority of models, derived from their mathematical and computational foundations, often clashes with their immunity to justified criticism (Saltelli et al. 2025). As emphasized in Section 2, numerous methods are effective in addressing these challenges. However, the authors argue that SA, with its “unsettling candor” (Leamer 2010), offers a unique hermeneutic framework—a key to translating the lines of code constituting a model into plain, interpretable language.

Determining the relevance of a model or suite of models often demands investigative work (Wynne 1984). When modelers proactively conduct sensitivity analyses, this work becomes anticipatory, fostering defensible and transparent practices. SA provides an “extra gear” for analyzing the model's internal dynamics to infer insights about the real‐world systems it represents (Morgan 2012). For this reason, the authors advocate integrating global SA into the model‐building process itself, rather than treating it as an ex post add‐on. This advice applies to all modelers, including those using similar methods such as multiverse analysis or perturbed physics ensembles, who can benefit from advances in SA.

The absence of SA in most higher education curricula—despite its critical importance in scientific modeling—requires attention. Addressing this gap requires equipping educators not only with appropriate tools and teaching materials but also with effective strategies to structure these resources to facilitate student learning. Such efforts can help bridge the divide between theoretical model development and practical validation, ultimately empowering future researchers to design models that are both mathematically rigorous and socially and scientifically accountable. This work represents a step in that direction, aiming to foster further dialogue and innovation in the pedagogy of SA.

## Funding

Andrea Saltelli gratefully acknowledges the European Union's Horizon Europe research and innovation program (project i4Driving with number 101076165).

## Conflicts of Interest

The authors declare no conflicts of interest.

## Data Availability

Data sharing not applicable to this article as no datasets were generated or analyzed during the current study.

## Endnotes

1 A discussion of Duhem's theses, and of their relevance to the difference between a theory and a model, can be found in the work of Morgan (2012), discussing how scientists and economists use models not merely as simplified representations of reality, but as autonomous, experimental spaces in which they construct, explore, and manipulate ‘model worlds’ to generate knowledge about the real world.

2 Elements of sociology of quantification can also be useful here, from Pierre Duhem to Mary S. Morgan (previous section), contrasting a syntactic view of models, that is, models as logical rules or mathematical equations, with a semantic view of models, where models can accommodate a plurality of different rules. The concept of SA as a hermeneutics of modeling can also be useful in this respect.

3 ECTS stands for European Credit Transfer and Accumulation System. One ECTS credit may represent approximately 25 to 30 hours of total student workload, of which, for example, 12 to 15 hours are spent in the classroom and the rest on independent projects or homework, as per https://education.ec.europa.eu
